# Prognostic significance of the number of tumors and aggressive surgical approach in colorectal cancer hepatic metastasis

**DOI:** 10.1186/1477-7819-12-155

**Published:** 2014-05-21

**Authors:** Kun-Ming Chan, Tsung-Han Wu, Chih-Hsien Cheng, Wei-Chen Lee, Jy-Ming Chiang, Jinn-Shiun Chen, Jeng-Yi Wang

**Affiliations:** 1 Department of General Surgery, Chang Gung Memorial Hospital at Linkou, Chang Gung University College of Medicine, 5 Fu-Hsing Street, Kwei-Shan Township, Taoyuan 33305, Taiwan; 2 Department of Colorectal Surgery, Chang Gung Memorial Hospital at Linkou, Chang Gung University College of Medicine, Taoyuan, Taiwan

**Keywords:** Colorectal cancer, Hepatic metastasis, Liver resection, Outcome

## Abstract

**Background:**

Although liver resection (LR) for colorectal cancer (CRC) hepatic metastasis is the best strategy to improve patient outcomes, there are considerable concerns regarding the recurrence of CRC after LR. In this study, we investigated the prognostic indicators associated with CRC recurrence after LR for hepatic metastasis.

**Methods:**

This is a retrospective review of patients who underwent curative LR for CRC hepatic metastasis between January 2008 and December 2012. The clinicopathological features and outcome parameters affecting prognosis were analyzed.

**Results:**

A total of 332 LRs with curative intent were performed in 278 patients, of whom 168 (60.4%) experienced CRC recurrence after the first LR, and 206 of the 332 LRs (62.0%) developed CRC recurrence. A preoperative serum carcinoembryonic antigen level greater than 100 ng/mL and four or more metastatic tumor nodules were independent prognostic factors for CRC recurrence after LR. The disease-free survival rate after LR was significantly associated with the number of metastatic nodules. The patients who underwent surgical resection for recurrent CRC had favorable outcomes, with a five-year overall survival rate of 65.2%.

**Conclusion:**

The number of metastatic tumors significantly affects the outcomes of patients who undergo LR for CRC hepatic metastasis, indicating that a novel therapeutic strategy for patients at high risk may be required. However, favorable long-term outcomes are achievable through aggressive treatment with surgical resection of the recurrent CRC.

## Background

The liver is the most common site of distant spread of primary colorectal cancer (CRC), and over 50% of patients will develop hepatic metastasis during the course of their disease [1,2]. Liver resection (LR) is believed to provide the only chance of curative treatment, and has largely improved the long-term outcomes of these patients if the metastatic CRC is confined to the liver [2-4]. With the introduction of multidisciplinary treatment and the advancement of surgical management and chemotherapeutic agents, the five-year survival rate following LR with curative intent for CRC hepatic metastasis has been reported to be up to 60% in recently published studies [5-7]. Nevertheless, despite the excellent results of surgical resection for metastatic CRC, it is estimated that more than half of the patients will still develop recurrence within two years [8,9].

CRC is a common gastrointestinal malignancy worldwide, and has recently been reported to be the most common cancer in East Asian countries. LR is increasingly being used as the standard practice for CRC hepatic metastasis as well. Although numerous previous studies have reported prognostic factors capable of predicting the outcomes for CRC patients undergoing LR for hepatic metastasis [5,10-12], predictors for CRC recurrence following LR remains entirely elusive. Moreover, despite a growing experience and literature, it is still an issue of great concern. In the current study, we retrospectively reviewed our experience with LR for patients with hepatic metastasis from CRC with the aim of providing additional information in terms of the factors associated with the prognosis of the patients undergoing LR, as well as the outcomes of CRC recurrence after LR.

## Methods

### Patients

This study included patients with CRC hepatic metastasis who underwent LR with curative intent between January 2008 and December 2012 at Chang Gung Memorial Hospital Linkou Medical Center (Linkou, Taiwan). A retrospective review of all medical records was performed with approval of the Institutional Review Board of Chang Gung Memorial Hospital. Data from the medical records including clinical characteristics, surgical management and outcomes were analyzed.

### Liver resection for hepatic metastasis

The clinical status of CRC and hepatic metastasis was thoroughly evaluated using appropriate imaging studies, including computed tomography (CT) scans of the abdominal and pelvic areas, and/or chest CT for all patients prior to surgery. Positron emission tomography (PET) or PET/CT was not routinely performed, but was occasionally performed for the patients who had equivocal conventional imaging study results to confirm occult metastasis if indicated. The treatment for CRC hepatic metastasis was decided by consensus of the members of the multidisciplinary committee, which was comprised of liver surgeons, proctologists, oncologists, radiologists and interventional radiologists. Treatment options mainly depended on the tumor’s characteristics and the patient’s physical condition, and liver resection was always the preferred treatment for patients with resectable hepatic metastasis. Resectability of hepatic metastasis with a curative intent required complete resection of all hepatic metastatic lesions, and preservation of a sufficient volume of liver with adequate vascular inflow and outflow. The extent of LR was defined on the basis of Couinaud’s classification. The patients with imaging evidence of concurrent unresectable extrahepatic metastasis were considered ineligible for LR.

### Follow-up after liver resection

After LR, postoperative adjuvant chemotherapy was recommended for all patients, unless the patient’s physical status was unsuitable for chemotherapy or they were unwilling to receive chemotherapy. The chemotherapeutic regimens mainly depended on the aggressiveness of the tumor characteristics, the patient’s physical condition, availability of the chemotherapeutic regimens, and affordability of the chemotherapy drugs. The chemotherapeutic options were mostly fluorouracil plus leucovorin and a combination of options, including oxaliplatin, irinotecan, bevacizumab and cetuximab. Additionally, all patients were regularly followed up and monitored for CRC recurrence by measuring serum carcinoembryonic antigen (CEA) levels and liver ultrasonography one month after LR and every three months thereafter. CT and/or magnetic resonance imaging (MRI) was performed at yearly intervals or whenever CRC recurrence was suspected. Disease recurrence was determined by a tissue sample from either a biopsy or surgical resection confirming CRC, and/or by serial imaging examinations. All patients were followed up until death or the end of the study period. The strategy for the treatment of recurrent CRC after LR was the same as that for the initial management of CRC, and depended on the consensus of the multidisciplinary committee.

### Statistical analysis

All statistical analyses were performed using SPSS statistical software version 17.0 (SPSS, Inc., Chicago, IL, USA) and Prism 5.0 (GraphPad Software, San Diego, CA, USA) for Windows. The end-point outcome measures were recurrence-free survival (RFS) and overall survival (OS). RFS was defined as the date of each LR to the date of detected CRC recurrence or the date of the last follow-up if there was no CRC recurrence. OS was defined as the date of the first LR to the date of death or the date of the last follow-up. Survival analysis was conducted using the Kaplan-Meier method. Variables were analyzed by multivariate analysis using a Cox regression proportional hazards model to identify the factors influencing RFS on the basis of each LR. An optimal cutoff value for continuous variables was determined by receiver-operating characteristic (ROC) curve analysis. All significant prognostic factors determined by univariate analysis and important clinical variables were then entered into multivariate analysis. Statistical significance was set at a *P-*value of less than 0.05.

## Results

### Clinical characteristics of the patients

A total of 332 LRs with curative intent were performed in 278 patients in this study. Of these patients, 186 (66.9%) were men and 92 (33.1%) were women, and the median age at the time of the first LR was 60.4 years (range, 29 to 88 years). After the first LR, the median follow-up period for the included patients was 23.8 months (range, 2 days to 108 months). Table 1 summarizes the clinical characteristics of the patients who underwent LR for CRC hepatic metastasis. The primary malignancy was located in the colon in 64% of the patients and 62% of the LRs. During follow-up, 168 patients (60.4%) experienced CRC recurrence after the first LR, and 206 of the 332 LRs (62.0%) developed CRC recurrence. Of the 168 patients with CRC recurrence, 61 (36.3%) underwent surgical resection for the CRC recurrence, and 74 (35.9%) surgical resections were performed for the 206 cases of CRC recurrence after LR. There were three cases of surgery-related mortality, and the mortality rates were 1.1% and 0.9% for all patients and the LRs, respectively.

**Table 1:** Clinicopathological characteristics of the patients who underwent liver resection for colorectal cancer hepatic metastasis

**Characteristics**	**Patients *n*** **= 278**	**Liver resections *n*** **= 332**
Age (years), median (range)	60.4 (29.1 to 88.0)	60.9 (29.1 to 88.0)
Gender		
Male	186 (66.9%)	225 (67.8%)
Female	92 (33.1%)	107 (32.2%)
Primary tumor location		
Colon	178 (64.0%)	206 (62.0%)
Rectum	100 (36.0%)	126 (38.0%)
Type of initial hepatic metastasis		
Synchronous	153 (55.0%)	153 (46.1%)
Metachronous	125 (45.0%)	179 (53.9%)
CRC recurrence after liver resection		
Yes	168 (60.4%)	206 (62.0%)
No	110 (39.6%)	126 (38.0%)
Surgical resection of CRC recurrence	61 (36.3%)*	74 (35.9%)*
Final patient status		
Alive and CRC free	128 (46.0%)	—
Alive with recurrent CRC	62 (22.3%)	—
Died of CRC	79 (28.4%)	—
Died of other causes	6 (2.2%)	—
Surgical mortality	3 (1.1%)	3 (0.9%)

CRC, colorectal cancer; * represents the percentage among CRC recurrence.

### Recurrence after liver resection of hepatic metastasis

Among the 332 LRs, the prognostic factors affecting CRC recurrence after LR were further analyzed and are summarized in Table 2. Univariate analysis identified the following five factors: preoperative serum CEA level, number of tumors, maximum tumor size, distribution of hepatic metastasis, and distance of resection margins. However, multivariate regression analysis of the prognostic factors showed that a preoperative serum CEA level ≥100 ng/mL (*P* = 0.0001, hazard ratio (HR) = 2.06) and four or more tumor nodules (*P* = 0.041, HR = 1.53) were independent prognostic factors of CRC recurrence following LR for hepatic metastasis.

**Table 2:** The clinicopathological factors affecting CRC recurrence of all liver resections for hepatic metastasis

**Factors**	**Univariate analysis**				**Multivariate analysis**	
	***n***	**Medium RFS (m)**	**HR, (95% CI)**	***P-*** **value**	**HR, (95% CI)**	***P-*** **alue**
Age (years)						
<55	108	12.9	1.16, (0.86 to 1.55)	0.327	—	—
≥55	224	14.7	1			
Gender						
Male	225	15.1	1	0.608	—	—
Female	107	11.2	1.08, (0.80 to 1.45)			
Primary tumor						
Colon	206	15.1	1	0.232	—	—
Rectum	126	12.0	1.11, (0.89 to 1.57)			
Serum CEA (ng/mL)						
<100	294	15.1	1	<0.0001	1	0.001
≥100	38	6.3	3.15, (1.83 to 5.44)		2.06, (1.36 to 3.11)	
Metastatic type						
Synchronous	153	14.8	1	0.757	1	0.744
Metachronous	179	13.1	0.95, (0.72 to 1.26)		1.05, (0.79 to 1.40)	
Number of tumors						
<4 nodules	285	15.4	1	0.001	1	0.041
≥4 nodules	47	8.3	2.22, (1.40 to 3.51)		1.53, (1.02 to 2.29)	
Maximum tumor size (cm)						
<5	267	13.6	1.39, (0.99 to 1.95)	0.058	1.40, (0.95 to 2.06)	0.088
≥5	65	21.7	1		1	
Distribution of metastasis						
Unilobar	239	15.9	1	0.025	1	0.399
Bilobar	93	9.7	1.43, (1.40 to 1.98)		1.16, (0.82 to 1.63)	
Extent of liver resection						
<3 segments	262	14.6	1	0.751	—	—
≥3 segments	70	13.1	0.95, (0.68 to 1.32)			
Resection margin (mm)						
<0.5	142	11.5	1.42, (1.08 to 1.88)	0.012	1.28, (0.95 to 1.71)	0.103
≥0.5	190	16.8	1		1	
Histologic grade						
Low-moderate grade	321	14.4	1	0.112	1	0.237
High grade	11	6.9	1.92, (0.86 to 4.28)		1.50, (0.77 to 2.94)	
Postoperative chemotherapy						
Fluorouracil	57	12.5	1.28, (0.85 to 1.93)	0.326	1.19, (0.80 to 1.76)	0.712
Oxaliplatin base	140	16.8	1		1	
Irinotecan base	103	12.8	1.34, (0.85 to 1.93)		1.19, (0.85 to 1.65)	
No	32	13.6	1.00, (0.60 to 1.67)		1.15, (0.68 to 1.95)	
Associated with bevacizumab						
Yes	46	17.0	1	0.496	—	—
No	286	13.5	1.15, (0.77 to 1.72)			
Associated with cetuximab						
Yes	6	7.4	1.80, (0.52 to 6.25)	0.352	—	—
No	326	14.4	1			
Chemotherapy cycles						
≥6	232	15.4	1	0.233	—	—
<6	100	9.7	1.21, (0.89 to 1.65)			

CEA, carcinoembryonic antigen; CI, confidence interval; CRC, colorectal cancer; HR, hazard ratio; RFS, recurrence free survival.

**Figure 1:**
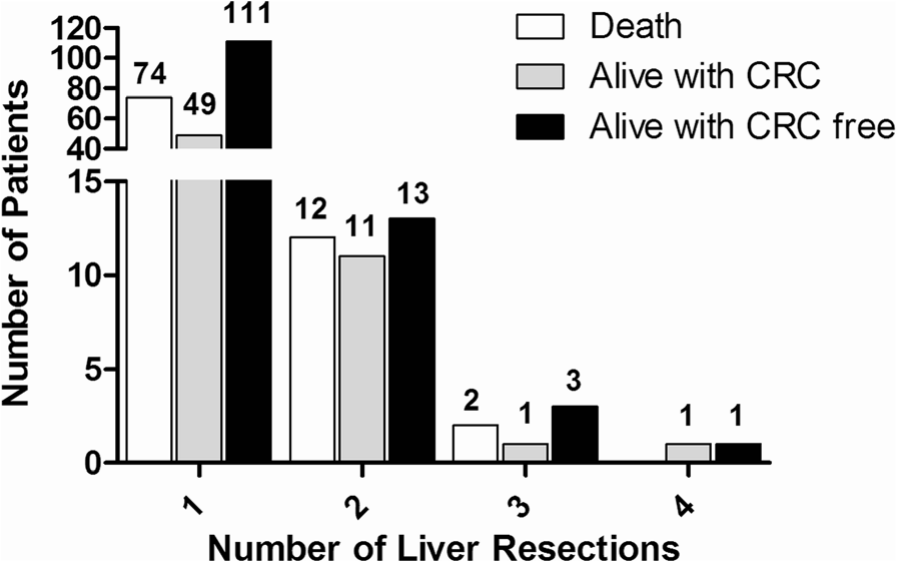
The final status of the patients related to the number of liver resections.

Of the 168 patients who developed CRC recurrence after LR, 206 cases of CRC recurrence, including 143 (69.4%) at a single anatomic site and 63 (30.6%) at multiple anatomic sites or systemic spreading, were detected. Table 3 summarizes the location of CRC recurrence and the surgical management; 74 surgical resections including 54 repeat LRs were performed for 61 patients accounting for 35.9% of the LRs with CRC recurrence and 36.3% of patients with CRC recurrence, respectively. With regards to the LRs, 44 patients received multiple LRs, and 2 of them underwent up to four LRs. Overall, 88 patients (31.7%) died, 62 (22.3%) were alive with CRC recurrence and 128 (46.0%) were alive without evidence of CRC at the end of the study period (Figure 1).

**Table 3:** CRC recurrence and surgical resection of recurrent lesions

**Recurrent features**	**CRC recurrence**^**†**^	Surgical resection^*^
**Number of patients**	168	61
**Location of recurrence**		
**Single anatomic site**		
Abdominal wall	2 (1.0%)	2 (2.7%)
Intraabdominal mass	16 (7.8%)	4 (5.4%)
Liver	90 (43.7%)	53 (71.6%)
Lung	29 (14.1%)	12 (16.2%)
Brain	3 (1.4%)	2 (2.7%)
Bone	3 (1.4%)	0 (0%)
**Multiple anatomic sites**		
Liver and lung	35 (17.0%)	1 (1.3%)
Systemic spreading	28 (13.6%)	0 (0%)
Total	206 recurrences	74 surgical resections

CRC, colorectal cancer; ^†^percentages represent the ratio among total recurrences;

*Percentages represent the ratio among total surgical resections.

### Survival analysis of the patients

**Figure 2:**
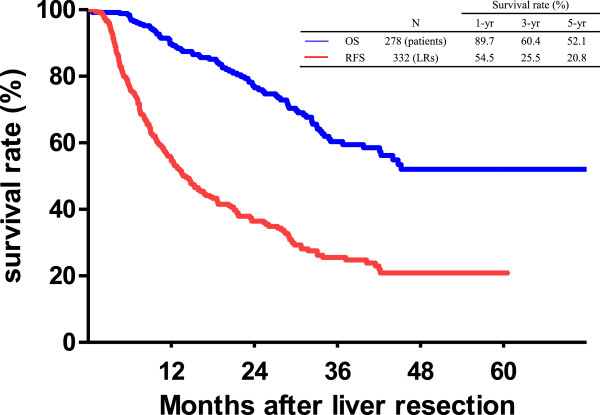
Kaplan-Meier cumulative survival curves of the patients who underwent liver resection (LR) for colorectal cancer (CRC) hepatic metastasis by recurrence-free survival (RFS) and overall survival (OS).

**Figure 3:**
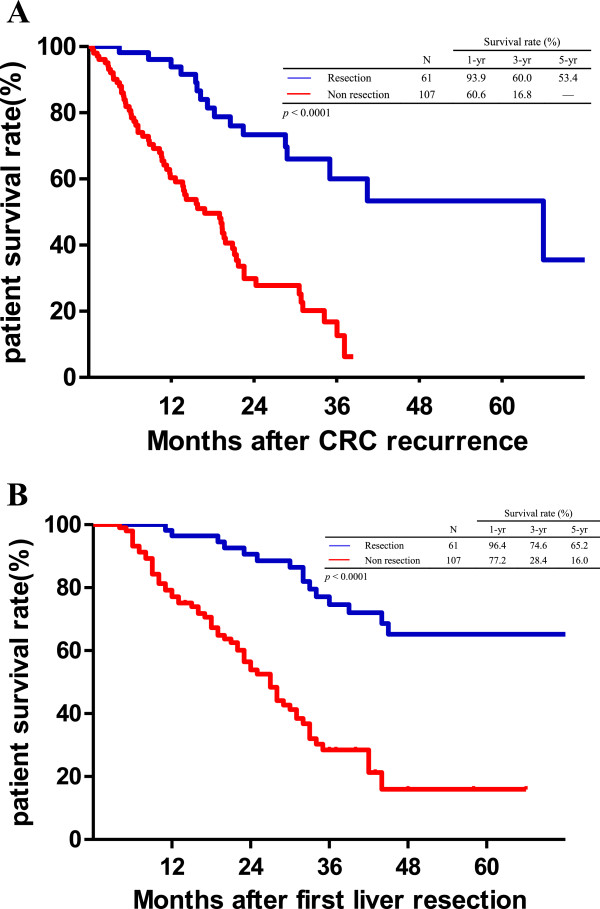
**Kaplan-Meier survival curves of the patients with colorectal cancer (CRC) recurrence after liver resection. A**. The patients who underwent surgical resection for CRC recurrence had a significantly better survival curve than those who did not undergo surgical resection for CRC recurrence. The three-year survival rates after CRC recurrence were 60.0% and 16.8% for the patients who did and did not undergo surgical resection of recurrent CRC, respectively (*P* <0.0001). **B**. The cumulative overall survival rates calculated from the first liver resection showed significant survival benefits for the patients who underwent surgical resection for CRC recurrence. The five-year overall survival rates were 65.2% and 16.0%, respectively (*P* <0.0001).

During the follow-up period, the median time of CRC recurrence after LR was 10.3 months, and the three- and five-year RFS rates were 25.5% and 20.8%, respectively. The median time of survival for all patients (*n* = 278) after the first LR was 23.7 months, with three- and five-year OS rates of 60.4% and 52.1%, respectively (Figure 2). Of those with CRC recurrence, the median survival after the first detection of recurrence was 14.4 months. The survival curve of the patients who underwent surgical resection for recurrent CRC was better than that of the patients who did not undergo surgical resection for recurrent CRC. The three-year survival rates after CRC recurrence were 60.0% and 16.8% for the patients with and without surgical resection, respectively (Figure 3A, *P* <0.0001). Moreover, the five-year OS rate of the patients who underwent surgical resection for CRC recurrence increased to 65.2% after the first LR, whereas the five-year OS rate of the patients who did not undergo surgical resection for CRC recurrence was only 16.0% (Figure 3B, *P* <0.0001).

**Figure 4:**
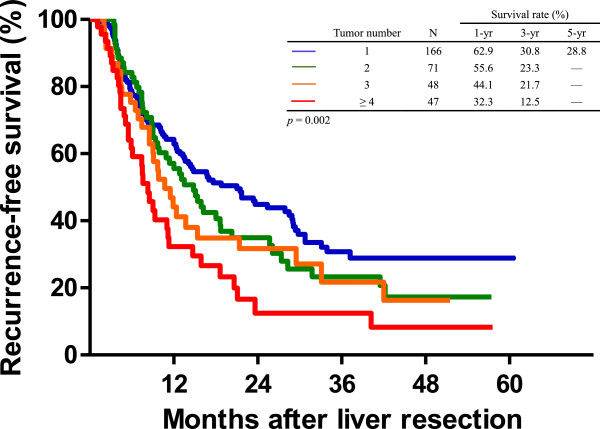
**The recurrence-free survival (RFS) curves of the patients after liver resection in terms of the number of hepatic metastatic tumors.** The patients with solitary metastatic tumors had the best RFS curve, and the RFS curves became worse as the number of tumor nodules increased (*P* = 0.002).

With regards to the number of metastatic tumors, the RFS of the patients was significantly associated with the number of metastatic nodules in the liver. The results showed that patients with a solitary metastatic tumor had a better survival curve, and the five-year RFS rate was 28.8%. As the number of tumor nodules increased, the actuarial RFS showed a significant decrease. Patients with four or more hepatic metastatic tumor nodules had the worst outcomes, with a five-year RFS rate of less than 10% (Figure 4, *P* = 0.002).

## Discussion and conclusion

Liver resection currently offers the best chance of survival and potential cure for patients with CRC hepatic metastasis, and numerous reports have demonstrated long-term survival benefits [5-7]. Along with advances in preoperative preparation, both resectability and OS of patients with CRC hepatic metastasis have shown remarkable improvements [13-15]. However, similar to patients who undergo surgical resection for primary cancer, CRC recurrence after LR for hepatic metastasis remains a concern worldwide. In this study, we found that the rate of cancer recurrence was still very high, and involved nearly 60% of the patients after LR for hepatic metastasis from CRC. However, the results also demonstrated that aggressive surgical resection for recurrent CRC was beneficial.

The treatment strategies regarding hepatic metastasis from CRC have changed along with advancements in systemic therapy in the last decade. Recent evidence has demonstrated that systemic chemotherapy contributes to improvements in OS in patients with hepatic metastasis from CRC [16-18], and that it is effective even as neoadjuvant therapy [19]. Although numerous factors probably affect prolonging patient survival, the use of chemotherapy clearly plays a key role. However, the importance of postoperative adjuvant chemotherapy was not found in this study. A possible explanation could be that the adjuvant chemotherapy regimens in our patients were not identical, and comparisons of patients grouped by differing chemotherapeutic regimens may have been limited by the small number of patients in each group. Unfortunately, no consensus currently exists regarding which therapeutic protocol is the best for the prevention of CRC recurrence after LR, and further studies to clarify the effect of specific postoperative adjuvant chemotherapy regimens on RFS are required.

A number of previous reports have shown that several prognostic factors affect the outcomes of patients who undergo LR for hepatic metastasis from CRC, and similar factors were also noted in this study. The presence of multiple hepatic metastatic tumors seems to be a very important risk factor for CRC recurrence after LR [11,15]. Patients with multiple hepatic metastases, and specifically more than four tumor nodules, have been reported to be associated with a poor prognosis, and many surgeons are therefore reluctant to perform LR for these patients. Nevertheless, several studies have indicated that long-term survival is achievable after LR for multiple CRC hepatic metastases [20-22]. Furthermore, the safety of hepatic resection and more effective adjuvant chemotherapy also support the concept of an aggressive surgical approach for patients with multiple CRC hepatic metastases. Due to the lack of other potentially curative alternatives, the presence of multiple hepatic metastases should not be considered as a contraindication for LR.

Surgical resection of metastatic lesions with curative intent is currently the treatment of choice for several malignancies [23-25], including for patients with recurrence after LR for CRC hepatic metastasis [26,27]. Our results also showed that surgical resection of isolated recurrent lesions was beneficial in selected patients who underwent LR for CRC hepatic metastasis. Although the prognosis of patients who are suitable for surgical resection may be better than for patients who are ineligible for surgical resection, an aggressive attitude in terms of surgical resection still seems to be beneficial. As shown in the current study, many of the patients were alive without CRC recurrence after multiple LRs. Moreover, sequential resection with curative intent for multiple metastases in various anatomic sites may also offer favorable survival outcomes.

Taken together, despite distant metastasis and the clinical indication as a terminal-stage cancer, CRC is one of the few malignancies for which patients with metastasis confined to a single organ may obtain long-term survival through multidisciplinary treatment. However, CRC recurrence remains a problem that affects more than half of the patients who undergo LR for hepatic metastasis. Due to the beneficial results of surgical resection for recurrent lesions, it is essential to regularly and frequently follow-up patients in the first few years after LR to ensure the early detection of CRC recurrence at a resectable stage. In addition, to achieve better long-term outcomes for patients with CRC and effectively treat hepatic metastasis, the development of a treatment protocol that involves surgery and chemotherapeutic regimens is indicated.

## Abbreviations

CEA: Carcinoembryonic antigen; CT: Computed tomography; CRC: Colorectal cancer HR, Hazard ratio; LR: Liver resection; OS: Overall survival; PET: Positron emission tomography; RFS: Recurrence-free survival.

## Competing interests

There was no financial support for this research and publication, and the authors have no conflicts of interest.

## Authors’ contributions

KMC carried out the study and wrote the paper. THW, CHC, WCL, JMC, JSC, JYW participated in the collection of data. All authors read and approved the final manuscript.
